# Anterior Chamber Biometric Parameters Associated With Intraocular Pressure Reduction After Phacoemulsification in Non-Glaucomatous Eyes With Open Angles

**DOI:** 10.7759/cureus.51500

**Published:** 2024-01-02

**Authors:** Deepayan Sarkar, Anshukita Anshukita, Samendra Karkhur, Bhavana Sharma, Saroj Gupta

**Affiliations:** 1 Ophthalmology, All India Institute of Medical Sciences, Bhopal, IND

**Keywords:** lens vault, anterior chamber parameters, non-glaucomatous eyes, phacoemulsification, intraocular pressure

## Abstract

Background: Decreased intra-ocular pressure after cataract surgery has been reported in eyes with and without glaucoma with variable magnitude. It is influenced by the anatomical structure of the anterior chamber. Preoperative evaluation of anterior chamber parameters can help to predict the change in intra-ocular pressure postoperatively.

Objective: The objective of the study is to evaluate intraocular pressure (IOP) change after uneventful phacoemulsification in non-glaucomatous eyes with open angles and its correlation with preoperative anterior chamber parameters measured by anterior segment optical coherence tomography (AS-OCT).

Methods: In this hospital-based prospective observational study, we included consecutive patients without glaucoma and open angles on gonioscopy who had undergone uncomplicated phacoemulsification with intraocular lens implantation (IOL). IOP and anterior chamber biometric parameters were measured preoperatively and compared with parameters obtained three months post-operatively by AS-OCT. The change in IOP and its relation to the parameters, including anterior chamber angle (ACA), anterior chamber depth (ACD), angle opening distance 500 μm anterior to the scleral spur (AOD500), anterior chamber width (ACW), lens vault (LV), and trabecular iris space area (TISA500) were evaluated. The main outcome measure was a change in IOP after phacoemulsification in normal eyes.

Results: Sixty-four eyes of 64 patients were enrolled. The mean patient age was 58.5 ± 9.4 years. The average IOP reduction was 2.43±1.64 mm of Hg from a preoperative mean of 16.77±2.54 mmHg three months after phacoemulsification surgery. The mean AOD500 increased significantly (0.440 ± 0.07 to 0.522 ±0.092) from preoperatively to three months postoperatively (p < 0.001). Preoperative lens vault and preoperative IOP had a strong positive correlation with the change in IOP at three months (r-value = 0.606; p-value <0.001) and (r-value = 0.73; p-value <0.001). There was a significant negative correlation between pre-operative TISA and AOD with change in IOP at three months (r-value = -0.545; p-value <0.001) and (r-value = -0.69; p-value <0.01).

Conclusion: Phacoemulsification surgery results in IOP reduction in non-glaucomatous eyes. Pre-operative IOP, lens vault, AOD, and TISA were significant predictors for IOP reduction.

## Introduction

Cataract surgery is one of the most commonly performed surgeries in ophthalmology [1]. Many studies have consistently shown that uneventful phacoemulsification with intra-ocular lens placement can deepen the anterior chamber and lower intraocular pressure (IOP) in glaucomatous and non-glaucomatous eyes [2-4].

Parameters such as preoperative IOP and anterior to the scleral spur (AOD) were significantly associated with reduced IOP after phacoemulsification [5,6].

Many other anterior chamber parameters have been proposed as independent predictors of IOP reduction after phacoemulsification, like anterior chamber angle (ACA), anterior chamber depth (ACD), and anterior chamber width (ACW) [5,7].

Pre-operative evaluation of these parameters with anterior segment optical coherence tomography (AS-OCT) provides clinicians with qualitative and quantitative information about the anatomical structure of the anterior chamber. The parameters that AS-OCT can quantitatively assess include AOD, ACA, ACD, ACW, trabecular iris space area (TISA), iris thickness, lens vault, etc. [7,8].

This study was done to prospectively evaluate the changes in the anterior chamber parameters after phacoemulsification in non-glaucomatous eyes and to evaluate the association of these parameters with changes in IOP post-operatively.

This study was previously presented as a poster at the All India Ophthalmological Society Annual Meeting on June 2, 2022.

## Materials and methods

In this prospective, observational study, patients with cataracts were consecutively recruited from the comprehensive service of a tertiary care center from December 2020 to November 2021. The study followed the tenets of the Declaration of Helsinki for biomedical research and was approved by the Institutional Human Ethics Committee (Number: LOP MD0079). Written informed consent was obtained from all participants before enrollment.

Inclusion criteria for patients scheduled for clear corneal phacoemulsification with foldable intraocular lens implantation included: age >18 years, presence of visually significant cataract, best corrected visual acuity (BCVA) worse than 20/40, an IOP of 20 mm Hg or less, and grade lll and lV angles on gonioscopy. Exclusion criteria included intraoperative complications and the need for adjunctive procedures; eyes with poor-quality AS-OCT images; eyes with glaucoma; previous intra-ocular surgery; intra-ocular pathology; and substantial corneal abnormalities.

Demographic data were recorded. Preoperative evaluation included the recording of BCVA, slit lamp, fundus examination, IOP measurement, gonioscopy, and ocular biometry. Gonioscopy was performed using a Posner 4-mirror gonioscopic lens by the same observer (D.S.) in a semi-dark room. Angles were graded in all four quadrants based on the Shaffer grading system. Indentation gonioscopy was done to rule out peripheral anterior synechia. IOP was measured using Goldmann applanation tonometry by a single observer (D.S.) one day before surgery and three months postoperatively. Three measurements were taken, and the average value was used for analysis.

The AS-OCT data were collected one day before surgery and three months after phacoemulsification using the anterior segment imaging premier module from CIRRUS HD-OCT 500, Carl Zeiss Meditec, Dublin, CA, USA, for all participants. Standard-resolution scans captured the nasal and temporal quadrants in one image, with the patient looking straight ahead with a good central corneal reflex. Several images were captured, and the image with the best quality without artifacts and visibility of the scleral spurs was chosen for analysis. Images in which the scleral spurs were not visible were removed from the analysis because quantitative measurements of the anterior chamber parameters by AS-OCT depend on correctly identifying the scleral spur [9].

Parameters measured in AS-OCT imaging were AOD 500, which is the perpendicular distance between a point 500 µm anterior to the scleral spur and the anterior iris surface, ACA is the angle between the point 500 μm from the scleral spur and the point on the anterior iris perpendicularly, with the apex at the iris recess, TISA 500 is the trapezoidal area bounded anteriorly by the AOD 500 line, posteriorly by the perpendicular distance between the scleral spur and the opposing iris, superiorly by the inner corneoscleral wall, and inferiorly by the anterior iris surface, ACD is the distance between the posterior corneal surface and the anterior surface of the crystalline lens, or IOL, measured at the center of the pupil, and ACW is measured as the line joining the temporal and the nasal scleral spurs.

The lens vault (LV) was measured as the perpendicular distance between the anterior lens pole and the horizontal line joining the temporal and nasal scleral spurs. Anterior chamber parameters measured on AS-OCT and the IOP were compared preoperatively and three months postoperatively.

All surgeries were performed under peribulbar anesthesia. Phacoemulsification was done via a 2.8-mm temporal clear corneal incision. A foldable IOL (Acrysof IQ SN60WF, Alcon Laboratories Inc., Fort Worth, TX, USA) was implanted in the bag. Patients with intraoperative complications were excluded from the study. All patients were re-examined three months postoperatively. IOP measurement and AS-OCT parameters were repeated as per the protocol.

Statistical Analysis: Data entry was done in Microsoft Excel 2010. Statistical analysis was done by IBM Corp. Released 2016. IBM SPSS Statistics for Windows, Version 24.0. Armonk, NY: IBM Corp. Continuous variables were summarized as mean ± standard deviation (SD). The mean difference of the data was calculated. Analysis of variances (ANOVA) and paired t-tests were used to compare mean preoperative and postoperative measurements. Relationships between anterior segment parameters and change in IOP were investigated using Pearson's correlation coefficient. A p-value of < 0.05 was considered statistically significant.

## Results

The study included 64 eyes of 64 patients undergoing phacoemulsification with IOL implantation in non-glaucomatous eyes with open angles. The mean age was 58.5 ± 9.4 years. Thirty patients were male, while 34 were female. The male-female ratio was 1:1.3. (Table 1).

**Table 1:** Baseline demographics and preoperative clinical parameters of study participants IOP: Intraocular pressure, n: Number, %: percentage

Characteristic	Value
Gender, n (%)	
Male	30 (46.9)
Female	34 (53.1)
Age (years)	
Range	25-75
Mean ± SD	58.5 ± 9.4
Preoperative IOP (mmHg)	
Range	12-20
Mean ± SD	16.77 ± 2.54
Lens vault (μm)	
Range	436-887
Mean ± SD	524 ± 64.2
Angle opening distance 500 (mm)	
Range	0.30-0.60
Mean ± SD	0.44 ± 0.07
Anterior chamber angle (degree)	
Range	23-45
Mean ± SD	31.62 ± 5.07
Trabecular Iris Surface Area 500 (mm^2^	
Range	0.12-0.53
Mean ± SD	0.19 ± 0.06
Anterior chamber depth (mm)	
Range	2.16-3.51
Mean ± SD	2.63 ± 0.29
Anterior chamber width (mm)	
Range	8.19-11.69
Mean ± SD	10.28 ± 0.61

Preoperative IOP ranged from 12-20 mmHg, and the average IOP was 16.77±2.54 mmHg. The average IOP three months after surgery was 14.22±1.75 mmHg, resulting in a 2.43±1.64 mmHg reduction in IOP from baseline and were found to be statistically significant (p <0.001). The mean AOD500, TISA500, ACA, and ACD values increased significantly after phacoemulsification with IOL implantation. No significant difference was found for ACW (Table 2).

**Table 2:** Comparison of ocular biometric parameters preoperatively and at three months postoperatively A p-value-value of 0.05 was considered statistically significant.

Parameters	Preop	Post-op (3 months)	Mean difference	p-value
Intraocular pressure (mm of Hg)	16.77±2.54	14.22±1.75	2.43±1.64	<0.001
Angle opening distance 500 (mm)	0.44±0.07	0.52±0.09	0.07±0.13	<0.001
Anterior chamber angle (degrees)	41.62±5.07	45.05±5.52	3.40±3.31	<0.001
Trabecular Iris Surface Área 500 (mm^2^)	0.19±0.06	0.25±0.07	0.05±0.02	<0.001
Anterior chamber depth (mm)	2.63±0.29	2.92±0.32	0.27±0.24	<0.001
Anterior chamber width (mm)	10.28±0.61	10.35±0.55	0.06±0.27	0.03

Pearson correlation analysis of the association between preoperative LV and IOP showed that preoperative lens vault value had a strong positive correlation with the change in IOP at postop three months (r-value = 0.606; p-value <0.001) and a significant positive correlation between pre-operative IOP and with IOP changes at postop three months (r-value = 0.73; p-value <0.001). There was a significant negative correlation between pre-operative TISA 500 and AOD 500 with change in IOP at three months (r-value = -0.545; p -value<0.001) and (r-value = -0.69; p -value=0.01). The pre-operative ACA and ACD were also negatively correlated with changes in IOP (r-value = -0.07; p-value = 0.67) and (r-value = -0.18; p-value = 0.28) (Table 3).

**Table 3:** Correlation of pre-operative ocular parameters with change in IOP postoperative at three months A p-value of < 0.05 was considered statistically significant. IOP: intraocular pressure, ACW: Anterior chamber width, ACD: Anterior chamber depth, TISA: Trabecular iris space area, AOD: Anterior to the scleral spur, ACA: Anterior chamber angle

Pre-operative lens vault	IOP change at three months
r value	0.606
p-value	<0.001*
Pre-operative IOP	
r value	0.73
p-value	<0.001*
Pre-operative AOD	
r value	-0.69
p-value	< 0.01*
Pre-operative ACA	
r value	-0.07
p-value	0.67
Pre-operative TISA	
r value	-0.545
p-value	<0.001*
Pre-operative ACD	
r value	-0.18
p-value	0.28
Pre-operative ACW	
r value	-0.18
p- value	0.28

There was a weak negative correlation between changes in ocular parameters at three months and changes in IOP at three months postoperative. It was statistically not significant (Table 4).

**Table 4:** Correlation of change in ocular parameters with change in IOP at three months postoperative A p-value of < 0.05 was considered statistically significant.

Anterior chamber parameters		IOP change at three months
Change in Angle opening distance (AOD 500) at three months	r value	-0.08
	p-value	0.62
Change in Anterior Chamber angle (ACA) at three months	r value	-0.26
	p-value	0.11
Change in TISA 500 at three months	r value	-0.09
	p-value	0.60
Change in Anterior Chamber Depth (ACD) at three months	r value	-0.21
	p-value	0.21
Change in Anterior Chamber Width (ACW) at three months	r value	-0.31
	p-value	0.06

## Discussion

In this prospective study, phacoemulsification with IOL implantation in non-glaucomatous eyes led to a mean decrease in IOP of 2.43±1.64 mmHg from a mean preoperative IOP of 16.77±2.54 mmHg; and it was found to be statistically significant (p<0.001). Preoperative ocular parameters that were significant predictors of IOP reduction were lens vault, angle-opening distance, trabecular iris space area, and preoperative IOP.

Many studies have shown that cataract surgery by clear corneal phacoemulsification with foldable intra-ocular lens implantation reduces IOP from 1.0 mmHg to 3.5 mmHg in nonglaucomatous eyes [2-4,10-13], and 1.0 mmHg to 5.5 mmHg in glaucomatous eyes [11,14-16].

A significant change in anterior segment biometric parameters has been reported after phacoemulsification based on anterior segment imaging by ultrasound biomicroscopy and AS-OCT [5-7,17,18]. In the present study, mean AOD 500, TISA 500, ACA, and ACD values significantly increased after phacoemulsification (p<0.001) due to the backward shift of the iris-lens diaphragm due to the replacement of the thick cataractous lens by the intraocular lens, which ultimately leads to deepening of the anterior chamber and widening of ACA [19].

The present study aimed to understand the preoperative parameters that can predict a reduction in IOP after phacoemulsification. We found a significant positive correlation between pre-operative IOP and change in IOP at three months postoperatively (r-value = 0.73; p-value <0.001).

A study by Hung et al., conducted on non-glaucomatous eyes, showed similar findings with pre-operative IOP as a predictor for the change of IOP after phacoemulsification. The extent of IOP reduction was positively related to preoperative IOP (r= 0.245, p=0.036). Other studies have also shown preoperative baseline IOP as the most consistent predictor for IOP reduction after cataract surgery in normal and glaucomatous eyes [3-6,20-22].

We found a significant positive relationship between preoperative lens vault and reduction in IOP at three months (r-value= 0.606; p-value<0.001), similar to the study by Hung et al. [5,7], in which preoperative lens vault correlated with the degree of IOP reduction and eyes with a higher lens vault and narrower trabecular iris space area had a greater angle-opening distance after cataract surgery.

Preoperative AOD and TISA were also significant predictors of reduction of IOP (p < 0.01 and p < 0.001). Bron et al. [23] concluded that pre-operative TISA 500 and AOD 500 were predictors of post-operative reduction in IOP, which agrees with our study’s results.

Several theories have been proposed to explain the mechanism of IOP reduction after phacoemulsification in normal eyes. It is likely due to the mechanical widening of the angle, leading to better aqueous humor outflow [5]. Another proposed theory is that there is an activation of an interleukin-1 alpha pathway in the trabecular meshwork secondary to ultrasound energy during phacoemulsification, which increases the facility of aqueous outflow. However, a randomized controlled trial comparing phacoemulsification surgery with small incision cataract surgery in normal eyes showed an equivalent reduction in IOP six months postoperatively, suggesting IOP reduction is more likely due to anatomical widening of the anterior chamber angle rather than an ultrasound-related pathway [24]. A third mechanism could be the expansion of Schlemm’s canal after phacoemulsification. The increase in the area and diameter of Schlemm’s canal after surgery correlated with the amount of IOP reduction and the increase of the anterior vault after phacoemulsification in eyes without glaucoma [25].

Study limitations: The study has certain limitations. First, the postoperative follow-up was limited to three months, which may not be sufficient for evaluating the long-term effect on IOP. However, several studies have reported that IOP reduction after phacoemulsification is sustained for many years [3,11,26,27]. Second, our sample size was relatively small. A study with a larger sample size would help provide further information on the anterior chamber biometric parameters and their correlation with IOP in eyes undergoing phacoemulsification.

This is the first study performed on the Indian population to prospectively evaluate the changes in the anterior chamber parameters after phacoemulsification in non-glaucomatous eyes and their correlation with the change in IOP, as well as whether the pre-operative biometric parameters can predict the IOP change post-operatively.

## Conclusions

Cataract surgery by phacoemulsification with intraocular lens implantation in non-glaucomatous eyes with open angles reduces IOP, which was found to be statistically significant. The preoperative anterior chamber parameters measured with AS-OCT showed a significant change after phacoemulsification at three months. The preoperative IOP is the most important predictor of IOP reduction after cataract surgery. There was a significant positive relationship between the preoperative lens vault and a decrease in IOP at three months.
